# Endoscopic Retrograde Cholangiopancreatography in Patients With Versus Without Prior Myocardial Infarction or Coronary Revascularization: A Nationwide Cohort Study

**DOI:** 10.7759/cureus.13921

**Published:** 2021-03-16

**Authors:** Harsh K Patel, Rupak Desai, Shreyans Doshi, Mohammad Haider, Neet Lakhani, Falah Abu Hassan, Rajkumar Doshi, Viveksandeep Thoguluva Chandrasekar

**Affiliations:** 1 Internal Medicine, Ochsner Clinic Foundation, New Orleans, USA; 2 Cardiology, Atlanta Veterans Affairs Medical Center, Decatur, USA; 3 Gastroenterology, Medical College of Georgia, Augusta University, Augusta, USA; 4 Internal Medicine, NewYork-Presbyterian Brooklyn Methodist Hospital, Brooklyn, USA; 5 Internal Medicine, Baroda Medical College, The Maharaja Sayajirao University of Baroda, Vadodara, IND; 6 Surgery, Princess of Wales Hospital, Bridgend, GBR; 7 Internal Medicine, University of Nevada, Reno School of Medicine, Reno, USA; 8 Gastroenterology, Mayo Clinic in Arizona, Scottsdale, USA

**Keywords:** endoscopic retrograde cholangiopancreatography, ercp, myocardial ischemia, myocardial infarction, coronary artery disease, percutaneous coronary intervention, coronary artery bypass grafting

## Abstract

Background

Endoscopic retrograde cholangiopancreatography (ERCP) can be associated with complications, including precipitation of peri-procedural myocardial ischemia. However, data regarding the trends and impact of previous myocardial infarction (MI) and/or percutaneous coronary intervention (PCI) or coronary artery bypass grafting (CABG) on ERCP outcomes remains unknown.

Methods

Using the National Inpatient Sample (2007-2014) and relevant ICD-9-CM codes, we identified adults who underwent ERCP with (Group 1) and without (Group 2) prior history of MI/PCI/CABG, and compared their demographics, comorbidities, and inpatient outcomes. Primary endpoints were inpatient mortality and post-ERCP complications. The secondary endpoints were discharge disposition, the mean length of stay, and total hospital charges.

Results

Of 1,374,773 ERCP procedures performed, 120,418 (8.8%) were performed in adult patients with a prior history of MI/PCI/CABG with an increasing trend from 2007-2014 (7.5% to 9.5%, p_trend_=0.022). Group 1 consisted of older, white, males compared to Group 2. Group 1 demonstrated a higher prevalence of all-cause mortality (1.7% vs. 1.5%, p<0.001), other cardiovascular comorbidities, post-ERCP cardiopulmonary complications (5.6% vs. 3.8%, p<0.001), sepsis (10.2% vs. 8.2%, p<0.001) and hemorrhage (1.5% vs.1.2%, p<0.001) as compared to Group 2. However, post-ERCP pancreatitis (14.1% vs. 15.4%, p<0.001) was lower in Group 1 without any difference in frequency of cholecystitis (0.4% vs. 0.4%, p=0.180). The mean length of stay was marginally higher in Group 1, without any difference in the hospitalization charges between the groups.

Conclusions

This nationwide study revealed higher inpatient mortality, sepsis, and hemorrhage in adult patients who underwent ERCP with a prior history of MI/PCI/CABG.

## Introduction

Endoscopic retrograde cholangiopancreatography (ERCP) has been increasingly performed in all age groups is the management of choice for many pancreaticobiliary pathologies. It helps to avoid the need for emergent highly invasive surgeries as it is a minimally invasive procedure with significantly less morbidity and mortality. However, there is limited data on the effect of the presence of comorbidities and outcomes in patients undergoing ERCP. Longer procedure time and the use of general anesthesia in patients with multiple co-morbidities and cholangitis puts them at higher risk of cardiovascular-related morbidity due to ERCP when compared to other GI endoscopic procedures [1,2]. Besides, increasing the use of ERCP as a therapeutic intervention compared to a diagnostic modality makes it essential to assess the appropriateness of its use in patients with a prior history of myocardial infarction (MI), percutaneous coronary intervention (PCI), or coronary artery bypass grafting (CABG).

There is a dearth of literature on the utilization of ERCP in patients with comorbid cardiovascular conditions. Little is known about those trends in recent years and how an ERCP affects the hospitalization outcomes in those patients. This national database study aimed to retrospectively identify the trends and compare outcomes in patients with a prior history of MI, PCI, or CABG who underwent inpatient ERCP as compared to those having no such comorbidities.

## Materials and methods

Data source

The National Inpatient Sample (NIS) 2007-2014, created by the Agency for Healthcare Research and Quality for Healthcare Cost and Utilization Project (HCUP), was queried to examine the study cohort [3]. The NIS is a publicly available all-payer inpatient dataset in the United States, which comprises a stratified sample of 20% nonfederal US community hospitals. Over 95% of the US population is embodied by this weighted dataset, which provides nationwide estimates of over 35 million hospitalizations each year. The NIS contains patient-level information including demographics, discharge diagnoses (one primary and 24 secondary diagnoses), in-hospital procedures (one primary and 14 secondary procedures), hospital-level features such as ownership, bed size, teaching status, urban/rural setting, and geographical region of hospitals. Resource utilization, such as mean length of stay (LOS), total hospitalization charges, and disposition are likewise consolidated into this dataset. An Institutional Review Board validation is not mandatory since the NIS dataset does not encase patient identifiers.

Study population

We examined ERCP-related hospitalizations in adult patients using ICD-9 CM procedure codes (51.10, 51.11, 52.13, 52.14, 52.21, 51.64, 52.92, 52.93, 52.94, 52.97, 52.98, 51.14, 51.88, 51.87, 51.86, 51.85, and 51.84) after excluding missing cases. A comorbid history of prior MI, PCI, or CABG cases was identified using the ICD-9 CM codes 412, V45.82, V45.81, respectively. Secondary discharge diagnoses were sought to evaluate ERCP-related complications by the relevant ICD-9 CM codes as validated and used in the previous studies [4]. We compared mortality outcomes in patients with a prior history of MI, PCI, or CABG as compared to those without such comorbidities

Study variables

We studied patient-level demographics including age, sex, race, type of admission, the day of admission, insurance payer type, median household income percentile, hospital-level characteristics including hospital ownership, bed size location/teaching status and region of hospital and hospitalization outcomes such as in-hospital mortality, LOS, total hospital charges and discharge disposition. We also incorporated cardiopulmonary comorbidities, post-ERCP pancreatitis, perforation, hemorrhage, and sepsis in our analysis from the secondary discharge diagnoses.

Outcomes

The primary endpoint was the trends in the ERCP procedures being performed in patients with a past medical history of MI, PCI, or CABG, and in-hospital mortality. The secondary endpoint was post-ERCP adverse events, mean LOS, hospital charges, and disposition of patients with and without a prior history of MI/PCI/CABG in patients undergoing ERCP.

Statistical analyses 

By applying sampling discharge weights to assess national estimates, we used the Pearson’s chi-square test and Student’s t-test to compare the categorical and continuous variables between the cohorts. Results were expressed in percentages and mean ± SD, respectively. We also evaluated predictors of in-hospital complications using a multivariable regression model that was adjusted for demographics, hospital characteristics, and baseline comorbidities. A two-tailed p-value <0.05 was used as the margin for the statistical significance. Statistical Package for the Social Sciences (SPSS) version 22 (IBM Corp., Armonk, NY) was used to perform all statistical analyses.

## Results

A total of 1,372,773 hospitalizations with (mean age 59.4 ± 19.8 years, 60.1% males) ERCP procedures were identified from discharge diagnosis between 2007 and 2014. Among them, 120,418 (8.8%) patients had a prior history of MI (3.7%), PCI (3.3%), or CABG (3.9%) (Group 1). The remaining cohort of patients was named Group 2.

**Figure 1:**
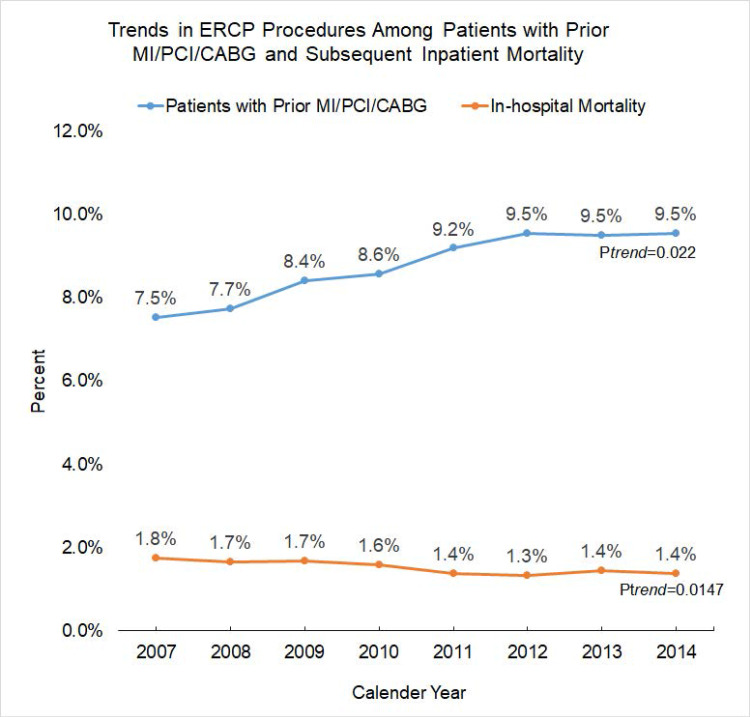
Trend in ERCP procedures among patients with prior MI/PCI/CABG and subsequent inpatient mortality ERCP: endoscopic retrograde cholangiopancreatography; MI: myocardial infarction; PCI: percutaneous coronary intervention; CABG: coronary artery bypass grafting

There was an increasing trend in ERCP use in Group 1 from 2007 to 2014 (7.5% to 9.5%, ptrend=0.022). At the same time, inpatient mortality in Group 1 decreased from 2007 to 2014 (1.8% to 1.4%, p_trend_=0.0147) (Figure 1). 

Table 1 presents the baseline characteristics of both groups. Group 1 cohort was relatively older (mean age 74 years vs. 58 years, p<0.001); had a higher proportion of the males (63.9%) compared to a higher proportion of the females (62.4%) in the Group 2 cohort (p<0.001); and mainly consisted of white (82%) patients. Patients in Group 2 had a higher proportion of patients of African American (9.4% vs. 5.4% p<0.001) and Hispanic (16.1% vs. 7.1%, p<0.001) ethnicity. There were statistically significant results but both groups were clinically comparable with respect to the type of admission (elective vs. nonelective), median household income, and bed size, the region as well as the location of the hospital.

**Table 1:** Baseline characteristics of patients undergoing ERCP with vs. without prior history of MI, PCI, or CABG P<0.05 indicates statistical significance. ERCP: endoscopic retrograde cholangiopancreatography; MI: myocardial infarction; PCI: percutaneous coronary intervention; CABG: coronary artery bypass grafting; HMO: health maintenance organization

Variable	Prior History of MI/PCI/CABG			p-Value
	No (Group 2) (n= 1,254,356)	Yes (Group 1) (n=120,418)	Overall (n=1,374,773 )	
Age (years) at hospitalization				<0.001
Mean±SD	58.0 ± 19.9	74.0 ± 11.2	59.4 ±19.8	
18-44 years	27.0%	1.0%	24.7%	
45-64 years	31.5%	18.7%	30.4%	
≥65 years	41.5%	80.3%	44.9%	
Sex				<0.001
Male	37.6%	63.9%	39.9%	
Female	62.4%	36.1%	60.1%	
Race				<0.001
White	66.7%	82.0%	68.1%	
African American	9.4%	5.4%	9.1%	
Hispanic	16.1%	7.1%	15.3%	
Asian or Pacific Islander	3.6%	2.5%	3.5%	
Native American	0.6%	0.6%	0.6%	
Others	3.5%	2.5%	3.4%	
Non-elective admission	87.3%	88.1%	87.3%	<0.001
Median household income national quartile				<0.001
0-25th	26.1%	24.4%	26.0%	
26-50th	25.6%	26.6%	25.7%	
51-75th	25.4%	25.0%	25.4%	
76-100th	22.8%	24.0%	22.9%	
Primary expected payer				<0.001
Medicare	42.3%	78.3%	45.5%	
Medicaid	13.0%	3.8%	12.2%	
Private including HMO	33.7%	14.8%	32.1%	
Self-pay/no charge/others	41.5%	16.4%	39.3%	
Bed size of hospital				<0.001
Small	9.9%	9.4%	9.8%	
Medium	24.2%	23.6%	24.1%	
Large	65.9%	67.0%	66.0%	
Location/teaching status of hospital				<0.001
Rural	5.6%	5.9%	5.6%	
Urban non-teaching	40.0%	40.3%	40.0%	
Urban teaching	54.5%	53.8%	54.4%	
Region of hospital				<0.001
Northeast	19.6%	20.9%	19.8%	
Midwest	22.1%	25.0%	22.4%	
South	35.2%	35.5%	35.2%	
West	23.0%	18.6%	22.7%	

Table 2 presents comorbidities in adult patients undergoing ERCP in Group 1 vs. Group 2. Comorbidities such as iron-deficiency anemia (21.8% vs. 17.0, p<0.001), congestive heart failure (20.2% vs. 5.8%, p<0.001), chronic pulmonary disease (21.6% vs. 13.3%, p<0.001), coagulopathy (8.3% vs. 6.1%, p<0.001), uncomplicated diabetes (33.7% vs. 17.6%, p<0.001), diabetes with complications (6.0% vs. 2.6%, p<0,001), hypertension (79.6% vs. 46.6%, p<0.001), dyslipidemia (57.2% vs. 22.2%, p<0.001), peripheral vascular disorder (12.8% vs. 3.3%, p<0.001), renal failure (18.3% vs. 7.4%, p<0.001) and valvular disorder (10.3% vs. 3.2%, p<0.001) were higher in patients in Group 1 compared to those in Group 2.

**Table 2:** Comorbidities in adult patients undergoing ERCP with vs. without prior history of MI, PCI, or CABG P<0.05 indicates statistical significance. ERCP: endoscopic retrograde cholangiopancreatography; MI: myocardial infarction; PCI: percutaneous coronary intervention; CABG: coronary artery bypass grafting

Comorbidities	Prior History of MI/PCI/CABG				p-Value
	No (n=1,254,356 )		Yes (n= 120,418)		
Alcohol abuse	51499	(4.1%)	3508	(2.9%)	<0.001
Deficiency anemias	213258	(17.0%)	26280	(21.8%)	<0.001
Congestive heart failure	72137	(5.8%)	24372	(20.2%)	<0.001
Chronic pulmonary disease	166597	(13.3%)	26002	(21.6%)	<0.001
Coagulopathy	76418	(6.1%)	9954	(8.3%)	<0.001
Diabetes, uncomplicated	221333	(17.6%)	40543	(33.7%)	<0.001
Diabetes with chronic complications	32499	(2.6%)	7182	(6.0%)	<0.001
Hypertension	583926	(46.6%)	95838	(79.6%)	<0.001
Hypothyroidism	134200	(10.7%)	17539	(14.6%)	<0.001
Liver disease	68177	(5.4%)	4956	(4.1%)	<0.001
Fluid and electrolyte disorders	358674	(28.6%)	37236	(30.9%)	<0.001
Metastatic cancer	46059	(3.7%)	3573	(3.0%)	<0.001
Dyslipidemia	278435	(22.2%)	68826	(57.2%)	<0.001
Smoking	264298	(21.1%)	34487	(28.6%)	<0.001
Obesity	156619	(12.5%)	12233	(10.2%)	<0.001
Peripheral vascular disorders	41291	(3.3%)	15444	(12.8%)	<0.001
Pulmonary circulation disorders	20206	(1.6%)	3930	(3.3%)	<0.001
Renal failure	92320	(7.4%)	22074	(18.3%)	<0.001
Valvular disease	40609	(3.2%)	12457	(10.3%)	<0.001

In a bivariate analysis of two groups (Table 3), patients in Group 1 had higher inpatient mortality (1.7% vs. 1.5%, p<0.001), cardiopulmonary events (5.6% vs. 3.8%, p<0.001), hemorrhage (1.5% vs. 1.2%, p<0.001) and sepsis (10.2% vs. 8.2%, p<0.001) compared to those in Group 2. However, Group 1 reported a lower rate of post-ERCP pancreatitis (PEP) (14.1% vs. 15.4%, p<0.001) compared to Group 2. There was no significant difference in presence of concomitant post-ERCP cholecystitis (0.4% vs. 0.4%, p=0.18) between the two groups. 

**Table 3:** Outcomes in adult patients undergoing ERCP with vs. without prior history of MI, PCI, or CABG P<0.05 indicates clinical significance. ERCP: endoscopic retrograde cholangiopancreatography; MI: myocardial infarction; PCI: percutaneous coronary intervention; CABG: coronary artery bypass grafting; SNF: skilled nursing facility; ICF: intermediate care facility

Outcomes	Prior History of MI/PCI/CABG				p-Value
	No (n=1,254,356 )		Yes (n= 120,418)		
In-hospital mortality	18888	(1.5%)	2099	(1.7%)	<0.001
Post-ERCP cardiopulmonary complications	47233	(3.8%)	6749	(5.6%)	<0.001
Post-ERCP pancreatitis	193275	(15.4%)	16976	(14.1%)	<0.001
Post-ERCP cholecystitis	4740	(0.4%)	485	(0.4%)	0.180
Post-ERCP perforation	1903	(0.2%)	127	(0.1%)	<0.001
Post-ERCP hemorrhage	15318	(1.2%)	1802	(1.5%)	<0.001
Post-ERCP sepsis	102593	(8.2%)	12282	(10.2%)	<0.001
Disposition of patient					<0.001
Routine	960775	(76.6%)	79345	(65.9%)	
Transfer to short-term hospital	27438	(2.2%)	2815	(2.3%)	
Other transfers (SNF, ICF, other)	117898	(9.4%)	17434	(14.5%)	
Home health care	122789	(9.8%)	18255	(15.2%)	
Length of stay mean [SD]	6.6 [SD 7.6] days		6.8 [SD 5.8] days		<0.001
Total hospital charges Mean	$60,142		$60,067		0.700

Patients undergoing ERCP in Group 1 were more likely to be discharged to short-term hospitals (2.3% vs. 2.2%, p<0.001), skilled nursing facility or intermediate care facility (14.5% vs. 9.4%, p<0.001), and home health care (15.2% vs. 9.8%, p<0.001) compared to Group 2. However, patients in Group 1 also demonstrated higher average LOS (6.8 days vs. 6.6 days, p< 0.001) but without any difference in the mean hospitalization charges ($60,067 vs. $60,142, p=0.700).

In a multivariable analysis for predictors of in-hospital complications (Table 4), patients with age>45 years, gender, races other than caucasian, admission at an urban facility or on a weekend, hospitals in the western United States, and prior comorbidities like chronic obstructive pulmonary disease, coagulopathy, congestive heart failure or valvular heart disease, fluid and electrolyte disorders, lymphoma, obesity, renal failure and solid tumor without metastasis were independently associated with higher odds of any complications (p<0.05). Patients admitted at a facility in the southern United States, when Medicaid was the primary payor, with known alcohol abuse, depression or psychosis, hypertension, and hypothyroidism were independently associated with lower odds of any complications during ERCP-related admission (p<0.05). 

**Table 4:** Multivariable predictors of any complication in ERCP-related hospitalizations P<0.05 indicates statistical significance. The multivariable model was adjusted for demographics, hospital characteristics, and baseline comorbidities. ERCP: endoscopic retrograde cholangiopancreatography; CI: confidence interval; OR: odds ratio

Predictors	Adjusted OR	95%CI		p-Value
		Lower	Upper	
Age (years) at admission				<0.001
18-44 years	Referent			
45-64 years	1.25	1.07	1.47	0.005
≥65 years	1.51	1.29	1.77	<0.001
Male vs. Female sex	1.08	1.04	1.11	<0.001
Race				
White	Referent			<0.001
African American	1.00	0.93	1.06	0.885
Hispanic	1.11	1.06	1.18	<0.001
Asian or Pacific Islander	1.18	1.08	1.28	<0.001
Native American	1.07	0.88	1.29	0.505
Others	1.32	1.21	1.44	<0.001
Non-elective vs. Elective admission	1.44	1.37	1.51	<0.001
Bed size of the hospital				0.478
Small	Referent			
Medium	1.02	0.97	1.07	0.504
Large	1.03	0.98	1.08	0.251
Location/teaching status of the hospital				<0.001
Rural	Referent			
Urban non-teaching	1.13	1.06	1.21	<0.001
Urban teaching	1.19	1.12	1.27	<0.001
Region of hospital				<0.001
Northeast	Referent			
Midwest	1.02	0.98	1.06	0.407
South	0.94	0.90	0.97	0.001
West	1.10	1.06	1.15	<0.001
Primary expected payer				<0.001
Medicare	Referent			
Medicaid	0.88	0.81	0.95	0.002
Private including health maintenance organization	1.02	0.97	1.07	0.408
Median household income national quartile for patient ZIP code				<0.001
0-25th	Referent			
26-50th	0.98	0.94	1.01	0.209
51-75th	0.99	0.95	1.03	0.621
76-100th	0.91	0.88	0.95	<0.001
Weekend vs. weekday admission	1.15	1.11	1.18	<0.001
Comorbidities				
Acquired immune deficiency syndrome	1.42	0.91	2.22	0.119
Alcohol abuse	0.89	0.81	0.97	0.005
Chronic blood loss anemia	1.05	0.91	1.23	0.490
Chronic pulmonary disease	1.07	1.04	1.11	<0.001
Coagulopathy	1.70	1.63	1.78	<0.001
Congestive heart failure	1.16	1.12	1.20	<0.001
Deficiency anemias	1.00	0.96	1.03	0.855
Depression	0.90	0.86	0.95	<0.001
Diabetes with chronic complications	0.97	0.92	1.03	0.357
Diabetes, uncomplicated	1.01	0.98	1.04	0.568
Drug abuse	0.95	0.82	1.10	0.487
Fluid and electrolyte disorders	1.39	1.35	1.43	<0.001
Hypertension	0.96	0.92	0.99	0.013
Hypothyroidism	0.95	0.92	0.99	0.020
Liver disease	0.93	0.87	1.00	0.048
Lymphoma	1.19	1.03	1.38	0.019
Metastatic cancer	1.05	0.97	1.14	0.247
Obesity	1.16	1.11	1.22	<0.001
Peptic ulcer disease excluding bleeding	1.22	0.88	1.68	0.235
Peripheral vascular disorders	0.96	0.92	1.00	0.034
Psychoses	0.81	0.74	0.90	<0.001
Pulmonary circulation disorders	1.06	0.98	1.14	0.135
Renal failure	1.14	1.10	1.19	<0.001
Rheumatoid arthritis/collagen vascular diseases	1.03	0.94	1.11	0.553
Solid tumor without metastasis	1.24	1.17	1.33	<0.001
Valvular heart disease	1.07	1.02	1.12	0.003

## Discussion

Cardiovascular morbidity, as well as mortality in patients undergoing endoscopic procedures with a recent history of acute coronary events, has been variable in different studies. There has been no consensus on their outcomes based on previous literature [5,6]. Overall, complications related to the endoscopy procedures have been reported to be the same in both the elderly and the young population [7]. However, most procedures, including EGD and colonoscopy, are relatively safer in patients having a risk of cardiovascular events when compared to ERCP [8]. The studies evaluating the safety and efficacy of ERCP in this patient population are scarce [9].

In our study, patients in Group 1 were older and had more pre-admission comorbidities when compared to patients in Group 2. A significantly higher prevalence of congestive heart failure (CHF) and hypertension in Group 1 patients can be traced to the low myocardial reserve as a result of the relevant cardiac events in the past. Thus, increased cardio-pulmonary complications, as well as inpatient mortality, could be multifactorial due to older age and other cardiovascular/ medical co-morbidities as evident from our multivariable regression analysis of predictors (Table 4). Patients after an acute coronary syndrome are advised to take an antiplatelet agent irrespective of the insertion of coronary stents, and most patients with a history of a PCI are on these medications. It can explain the higher incidence of hemorrhage in Group 1 patients, as some patients may not be able to come off these medications prior to the procedure. Sphincterotomy is an ERCP intervention with a relatively higher rate (up to 30% of cases with immediate bleeding) of post-ERCP bleeding and pancreatitis, which in turn can worsen the hypotension and increase the risk of myocardial ischemia [10]. Thus, it is advised to adhere to guidelines and consult cardiology for managing anticoagulation during ERCP in such patients [11]. 

Current literature lacks the practice trends with regards to the concern of increased mortality from ERCP in patients with a prior history of MI/PCI/CABG, which our study has addressed. We divided the available dataset into two groups of patients- one with a previous history of MI/PCI/CABG (Group 1, n=120,418) and one with no such prior history (Group 2, n=1,254,356). The results showed a rising trend of ERCP in patients with a previous history of MI/PCI/CABG from 2007 to 2014. It, however, did not result in increased in-patient mortality in such patients. Moreover, all-cause inpatient mortality was reduced from 1.8% in 2007 to 1.4% in 2014. The exact reason for this trend is not known but could be partly due to advancements in therapeutic endoscopy and appropriate rationalization of indications for which the procedure is being performed. As expected overall mortality in Group 1 was higher when compared to Group 2, which could be due to the multiple co-morbid statuses of these patients (1.7% vs. 1.5%, p<0.001).

Koh et al. retrospectively studied the complications of ERCP in 50 patients with a history of acute coronary syndrome, which also included 26/50 patients with coronary stents [12]. The incidence of PEP was 12%, and that of immediate bleeding was 14%. There was one event of excessive post-ERCP delayed bleeding leading to cardiac ischemia and ultimately resulting in death. However, no other adverse cardiopulmonary complication was reported in any patient. In our study, 3.3% and 3.9% of patients who underwent ERCP had a history of PCI and CABG, respectively, and PEP occurred in 15.4% of patients in Group 2. To our knowledge, it is the first study to report the incidence of post-ERCP pancreatitis in such a large number of patients with a history of PCI or CABG.

The biggest strength of our study was the sample size in both groups. Previous studies have lacked the assessment of the LOS and subsequent hospital charges. Our results show a higher mean LOS in Group 1 compared to Group 2 patients; however, it did not result in increased hospital charges. However, Group 1 patients were more likely to discharge to a skilled nursing/intermediate care facility or home health care possible because of older age, polypharmacy, and co-morbidities. We also performed a detailed multivariable regression analysis to identify predictors of complications in these patients. 

Nojkov et al. previously reported on the outcomes of therapeutic ERCP in 19 patients in whom the procedure was conducted within 30 days of an acute attack of MI or unstable angina [13]. It was suggested that therapeutic ERCP could be safely performed for such patients within the first 30 days but preferably only for emergent indications and only after patient optimization. However, they recommended that diagnostic ERCP should be avoided as long as magnetic resonance cholangiopancreaticography (MRCP) can be performed in such patients.

Our study has a few limitations. Limitations associated with the NIS dataset precluded our ability to evaluate the outcomes by stratifying them based on the duration and association between cardiopulmonary events and the ERCP in Group 1 patients. It limits our understanding of the application of the results specific to patients who had ERCP <30 days or >30 days after MI/PCI/CABG. We also could not report laboratory values in these patients as well as recurrence of acute coronary syndrome during or post-ERCP as a separate outcome in these patients. It has been assessed by few retrospective studies in the past, which recommend monitoring the levels of troponin in such patients as it correctly identifies the ones at risk of having recurrent ischemia [12,14].

## Conclusions

In this nationwide retrospective analysis, we determined a rising trend in the utilization of ERCP procedures among patients with prior MI/PCI/CABG. Adult patients undergoing ERCP with a previous history of MI, PCI, or CABG had higher inpatient mortality, cardiopulmonary events, sepsis, and hemorrhage compared to those without this history. Future large prospective studies on outcomes of ERCP are warranted to define the impact of prior infarction or revascularization and timing prior to ERCP.
